# Microfluidic liquid sheets as large-area targets for high repetition XFELs

**DOI:** 10.3389/fmolb.2022.1048932

**Published:** 2022-12-09

**Authors:** David J. Hoffman, Tim B. Van Driel, Thomas Kroll, Christopher J. Crissman, Elizabeth S. Ryland, Kacie J. Nelson, Amy A. Cordones, Jake D. Koralek, Daniel P. DePonte

**Affiliations:** ^1^ SLAC National Accelerator Laboratory, Menlo Park, CA, United States; ^2^ Stanford Synchrotron Radiation Lightsource, SLAC National Accelerator Laboratory, Stanford University, Menlo Park, CA, United States; ^3^ United States Military Academy, West Point, NY, United States; ^4^ SLAC National Accelerator Laboratory, Stanford PULSE Institute, Menlo Park, CA, United States

**Keywords:** microfluidics, x-ray, structural biology, devices, instruments, samples, liquid microjets, spectroscopy

## Abstract

The high intensity of X-ray free electron lasers (XFELs) can damage solution-phase samples on every scale, ranging from the molecular or electronic structure of a sample to the macroscopic structure of a liquid microjet. By using a large surface area liquid sheet microjet as a sample target instead of a standard cylindrical microjet, the incident X-ray spot size can be increased such that the incident intensity falls below the damage threshold. This capability is becoming particularly important for high repetition rate XFELs, where destroying a target with each pulse would require prohibitively large volumes of sample. We present here a study of microfluidic liquid sheet dimensions as a function of liquid flow rate. Sheet lengths, widths and thickness gradients are shown for three styles of nozzles fabricated from isotropically etched glass. In-vacuum operation and sample recirculation using these nozzles is demonstrated. The effects of intense XFEL pulses on the structure of a liquid sheet are also briefly examined.

## 1 Introduction

The use of liquid microjets has enabled great advances in solution-phase X-ray and ultraviolet spectroscopies and scattering methods by providing a constantly refreshing, windowless, vacuum-stable target (Smith and Saykally, 2017). The self-replenishing nature of the microjets is critical for their use in experiments with strongly ionizing radiation, as the sample of interest (or its solvent) can be rapidly degraded by continual exposure to ionizing radiation (Henderson, 1995; Neutze et al., 2000). An intense, highly focused pulse from an X-ray free-electron laser (XFEL) can even destroy the macroscopic microjet structure (Stan et al., 2016; Grünbein et al., 2021). While traditional microjets are suitable for most current-generation XFEL applications, the advent of next generation, high-luminosity XFELs requires additional consideration for ways to avoid damage to sample, solvent, or liquid microjet structure.

Liquid sheet jets have demonstrated substantial additional benefits over the traditional microjets for XFEL applications of solution-phase samples (Ekimova et al., 2015; Galinis et al., 2017; Koralek et al., 2018; Menzi et al., 2020; Nunes et al., 2020; Crissman et al., 2022). Liquid sheets provide a target which can be hundreds of microns to several millimeters wide while having thicknesses on scales from tens of microns to tens of nanometers. The short path length is particularly important for soft X-ray and electron transmission (Nunes et al., 2020) where a sufficiently thin cylindrical target would typically be much smaller than the probe beam diameter. Using a wide sheet target not only allows for better use of incident flux, but allows the beam to be spread out more, reducing intensity to avoid damage to the liquid sheet structure or the sample itself. If the sample is to be optically pumped, flat liquid sheets are also desirable to reduce lensing.

**FIGURE 1:**
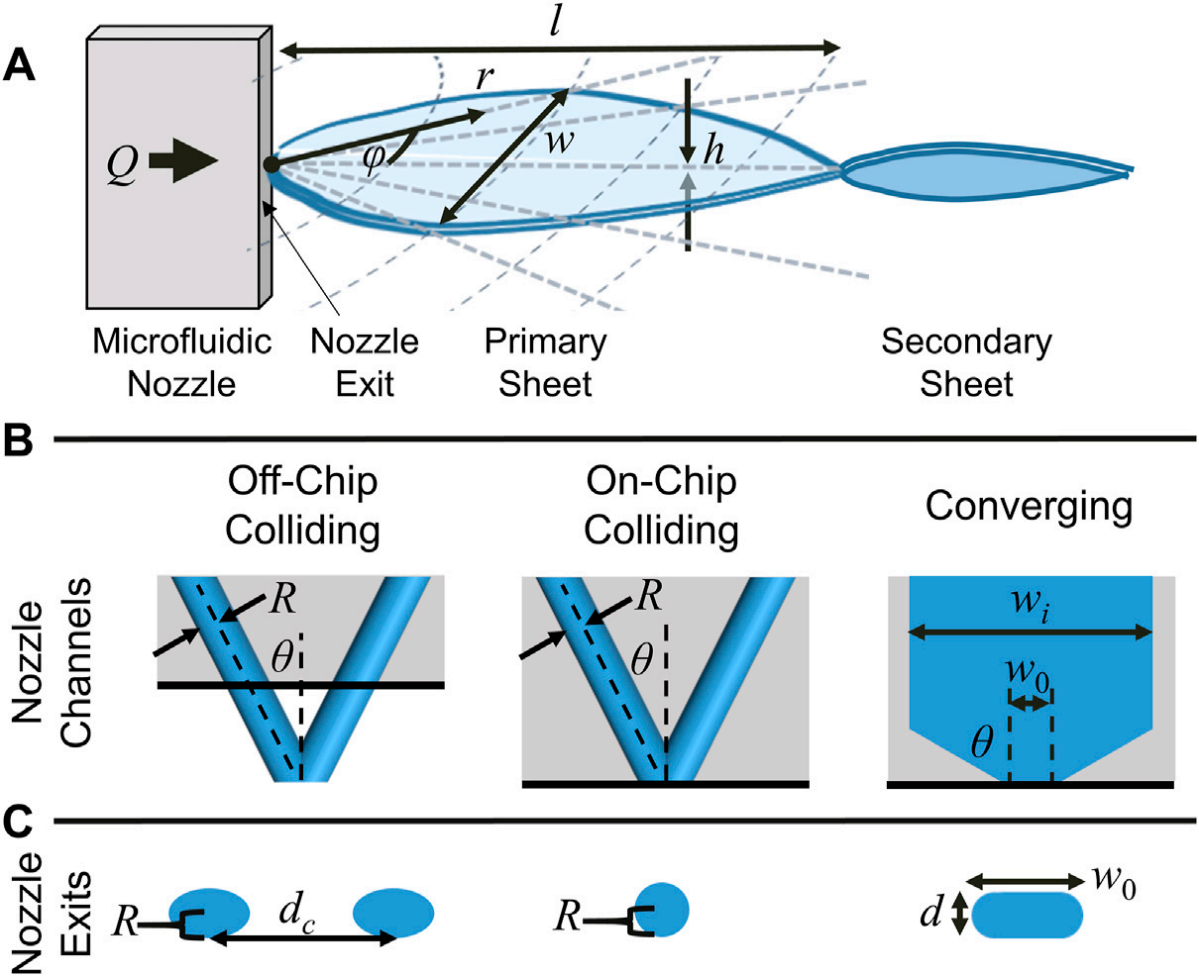
**(A)** Schematic illustration of a liquid sheet produced by a microfluidic nozzle and its principal dimensions. The liquid flow (*Q*) is from left to right. Momentum transfer at (or near) the nozzle exit creates a collision point which produces a flat liquid sheet perpendicular to the nozzle channels and bounded by cylindrical liquid rims. Surface tension brings the outer rims back together to a node, where a second, smaller sheet is produced orthogonal to the first. Subsequent sheets are smaller and orthogonal to the preceding sheet. **(B)** The three types of channel geometries examined in this work and their principal dimensions. **(C)** The nozzle exits for the three types of channel geometries.

Thin liquid sheets (illustrated in Figure 1A) can be produced using a variety of techniques, although the best studied method involves the collision of two liquid jets (e.g., Figure 1B, left) (Taylor, 1960; Hasson and Peck, 1964; Choo and Kang, 2001; Choo and Kang, 2002; Bush and Hasha, 2004). When the jets collide, momentum transfer results in the formation of a thin liquid sheet bounded by thick cylindrical rims in the plane orthogonal to the original jets. Surface tension brings the cylindrical rims back together at a node, where another, smaller sheet is generated orthogonal to the first. Closely analogous structures can be generated by means of a similar momentum transfer using a single converging channel (Ha et al., 2018; Crissman et al., 2022) (Figure 1B, right) or impinging gas jets on a cylindrical jet (Koralek et al., 2018).

This work examines the dimensions and scalings of liquid sheets produced by isotropically etched microfluidic nozzles that have been developed for XFEL applications (Koralek et al., 2018; Crissman et al., 2022; Hoffman et al., 2022), and the outlook for using these sheets as large-area targets for next-generation XFELs. Three distinct nozzle geometries were examined and compared: a traditional round jet colliding geometry (“off-chip colliding”, Figure 1B, left), a colliding jet geometry where the collision point is at the chip exit (“on-chip colliding”, Figure 1B, center), and a converging channel geometry (Figure 1B, right). The variation in thickness both across the sheet and as a function of flow rate is examined, as well as the length and width of the produced sheets. All three nozzles produced similar sheets which followed similar hydrodynamic scalings. The converging nozzles were operated in ambient air and vacuum. To mitigate the issue of the comparatively high flow rates used to produce sheets as compared to cylindrical jets, a closed loop recirculation procedure for sheets operating in vacuum was also developed. Finally, the structural damage induced by a strong XFEL pulse on a liquid sheet was examined.

## 2 Materials and methods

### 2.1 Microfluidic nozzles

Both the converging and colliding nozzles used in this work have been described in prior publications (Koralek et al., 2018; Crissman et al., 2022). The nozzles are produced in glass “chip” format by standard wet etch lithography methods by Micronit Ltd. The three channel geometries examined in this work are shown schematically in Figures 1B,C. For the converging nozzles examined, convergence angle (*θ*) of 60° was used with an initial channel width (*w*_*i*_) of 1 μm and two channel depths *(d*): 20 and 100 μm. For the colliding nozzles examined, the two channels used a *θ* = 40° colliding angle and had channel radii (*R*) of either 20 or 25 μm. The etch and bond lithography process results in slightly elliptical channels for the colliding nozzles and rounded channel edges for the converging nozzles, as illustrated in Figure 1C.

The converging nozzle exit face was polished with a 3 μm lapping film to diminish surface roughness around the nozzle exit which can negatively affect the surface quality of the liquid sheet (Crissman et al., 2022). For the converging nozzles, polishing will also increase the nozzle aperture’s width (*w*_0_), which has a strong effect on the thickness of the produced liquid sheets and a weaker effect on the sheets’ dimensions. Polishing the colliding chip exit face sets the location of the jet collision point relative to the face of the nozzle (increasing *d*_*c*_), ranging from within the chip (“on-chip colliding” geometry) to several hundred μm outside of the chip (“off-chip colliding” geometry). These changes had weak effects on the sheets’ dimensions but noticeably affected the stability of the sheet jets, with the on-chip colliding nozzles producing more stable structures.

### 2.2 Sheet jet operations and measurements

For the sheet dimension measurements, deionized liquid water was used. A high pressure liquid chromatography (HPLC) pump connected to a pulsation dampener was used to supply flow at rates from 1 to 50 ml/min depending on sheet type and size. For all measurements of sheet dimensions, the liquid sheets were oriented vertically with the liquid jet flowing downward, although orientation of the flow did not have noticeable impacts on the sheet geometry.

For measuring the sheet jet width, length and thickness profile, the sheet was placed in the field of view of a microscope calibrated to a grid distance standard. The nozzle was mounted to a rotary stage with the rotational axis along the sheet's long axis to align the sheet normal to the camera axis, typically 90 degrees to the nozzle plane. For thickness measurements, the sheet was illuminated with a white LED at a 30° angle of incidence. The specularly reflected thin film interference pattern could then be used to quantitatively describe the thickness of the sheet, as has been described in detail in prior publications (Choo and Kang, 2001; Koralek et al., 2018; Menzi et al., 2020; Crissman et al., 2022; Hoffman et al., 2022).

### 2.3 In-vacuum sample recirculation

For recirculating relatively small sample volumes while jetting in vacuum, a recirculation setup was constructed. A schematic diagram of the recirculation setup can be found in the Supplementary Material. In the vacuum chamber, the jet is aimed into a conical heated catcher (Innovative Research Solutions) with a 500 μm diameter hole connected to ¼ stainless steel tube. This tube is connected to a small pressure vessel that was cooled to 2°C. Gravity and the vapor pressure gradient between the heated catcher and chilled pressure vessel allows the liquid sample to passively collect in the chilled pressure vessel. The bottom of the chilled pressure vessel is connected to a valve which is connected to a second pressure vessel. The second vessel serves as an interlock between the vacuum chamber and the atmosphere. The interlock is pumped to vacuum, and a valve is opened between the two vessels to allow the liquid to fall under gravity into the interlock. The valve closes, and a second valve is opened at the bottom of the interlock which feeds back to the sample reservoir. The interlock vessel is simultaneously back filled with nitrogen to empty the interlock to the reservoir after which the nitrogen is evacuated using a scroll pump. This cycle repeats every few minutes to enable sample recirculation.

### 2.4 Evaporative loss

Evaporative loss for the recirculation system was studied by monitoring sample concentration of 8.5 mM solution of Tris(bipyridine)ruthenium(II) chloride ([Ru(bpy)_3_]^2+^ 2Cl^−^, “Rubipy”), a common inorganic dye (Kalyanasundaram, 1982). The solution concentration was monitored using a NanoDrop UV-Vis spectrophotometer (Thermo Scientific) in 10 μl aliquots taken from the sample reservoir. The concentration of the solution could be directly determined from the UV-Vis spectra through Beer’s law: A=εcl , where the absorption of the solution at a given wavelength, *A*, is given by the molar absorption coefficient of Rubipy for that wavelength, *ε*, the concentration of the solution, *c*, and the optical path length *l*. Concentrations were determined based on the absorption of Rubipy at 452 nm using the literature value for the absorption coefficient of 14,600 cm^−1^ M^−1^ (Kalyanasundaram, 1982).

### 2.5 Monitoring sheet damage from X-ray free electron lasers pulses

To observe the impacts of intense hard X-ray XFEL pulses on the sheet jets, a sheet from a converging nozzle (*d* = 100 μm, *w*_0_ = 578 μm) was monitored at the X-ray Correlation Spectroscopy (XCS) instrument at the Linac Coherent Light Source (LCLS). The sheet was imaged with a single-shot Alvium camera triggered to the X-ray beam and illuminated with a short 3 μs 780 nm LED pulse. The sheet was probed and sampled at 120 Hz to allow for visualization of the jet stability and jet explosion resulting from single X-ray pulses as the sheet advances. The hard X-ray pulses had pulse energies of ∼0.5 mJ at a 9.5 keV photon energy with a 40 fs duration. These pulse parameters resulted in clear jet explosions when the X-ray beam was focused (∼5 μm spot size). The pulsed illumination scheme allowed for the scanning of the illuminated delay and imaging of the visible sheet damage at high intensities.

## 3 Results

### 3.1 Sheet thickness

#### 3.1.1 Background

The thickness of the liquid sheets created by colliding or converging nozzles has been examined by many prior publications (Taylor, 1960; Hasson and Peck, 1964; Bush and Hasha, 2004; Ekimova et al., 2015; Galinis et al., 2017; Ha et al., 2018; Menzi et al., 2020; Yin et al., 2020; Crissman et al., 2022; Hoffman et al., 2022). For both styles of nozzles, useful relations can be derived from Taylor’s assumption that the liquid sheet flows essentially radially outward from the collision point at constant velocity, *u*_0_ (Taylor, 1960). From conservation of mass, this implies that

$${hru}_{0}=Q\left(\varphi \right)$$ (1)

where h is the thickness of the sheet, *r* is the radial distance from the collision point, and $Q\left(\varphi \right)$ is the angular flux of the liquid. Ideally, both $Q\left(\varphi \right)$ and $u_{0}$ are linear in flow rate, Q . These contributions cancel, which gives the well-known result that sheet thickness is independent of flow rate. Instead, the sheet thickness depends only on the nozzle geometry and the radial distance from the collision point. Different nozzle geometries will generally result in different $Q\left(\varphi \right)$ and *u*_0._

As a result, the product *hr* (“thickness scaling”) is invariant across the sheet for a given angle *φ*, and can be used to describe the sheet thickness profile. Previous work (Crissman et al., 2022; Hoffman et al., 2022) examining the thickness profile of these sheets along their centerline (*φ* = 0°) found that the thickness of the sheets produced by these nozzles follow the expression:

$$h=\frac{cA}{r}$$ (2)

where c is a dimensionless constant given by the nozzle geometry and *A* is the cross-sectional area of the nozzle channels with *A* = *dw*_0_ for the converging nozzles and *A* = 2*πR* ^2^ for the colliding nozzles. The converging nozzle area is slightly overestimated by approximating it as a rectangle, but the error is less than 5% for the nozzle dimensions used in this work. In recent work, *c* was found to be 0.84 for these converging nozzles (Crissman et al., 2022) and (neglecting the mild flow rate dependence discussed below) 0.46 for these colliding nozzles (Hoffman et al., 2022).

#### 3.1.2 Angular variation of thickness

**FIGURE 2:**
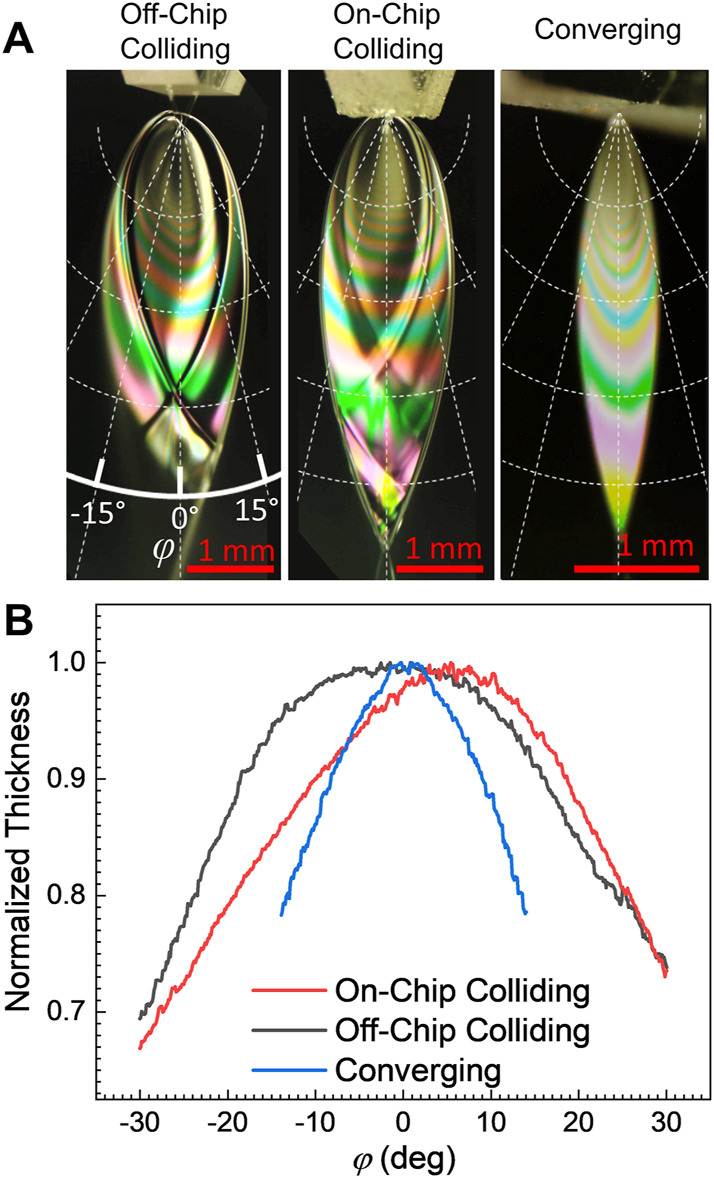
**(A)** Example white light thin film interference patterns for the three nozzle types examined in this work. Each colored fringe corresponds to a particular liquid sheet thickness, which then corresponds to a contour of equal thickness in the sheet. As the liquid in the sheet flows radially outward from the collision point, the sheet becomes flatter with distance from the nozzle. **(B)** Normalized sheet thickness profiles as a function of angle *φ*. The non-normalized sheet thickness varies with the radial distance from the collision point per Eq. 1. The on-chip colliding nozzle produced the flattest sheet, while the converging nozzle produced the sheets with the greatest thickness variation.

The nozzle geometry is known to have a strong impact on the angular liquid flux, *Q*(*φ*), which can then have a strong impact on the variation in thickness laterally across the sheet through Eq. 1 (Taylor, 1960; Hasson and Peck, 1964; Choo and Kang, 2001; Choo and Kang, 2002; Bush and Hasha, 2004). While it is challenging to derive useful analytic expressions for *Q*(*φ*) from the nozzle geometry (Choo and Kang, 2001; Bush and Hasha, 2004), it can be readily measured from the sheet’s thickness profile using Eq. 1. The thickness profiles of an off-chip (*R* = 25 μm, *d*_*c*_ = 700 μm) and on-chip colliding nozzle (*R* = 25 μm) and a converging nozzle (*w*_0_ = 100 μm, *d* = 20 μm) were examined using the reflected white light thin-film interference pattern from the sheet (see *Materials and methods* section). Microscope images of the sheet reflections can be seen in Figure 2A. Each distinct colored fringe in the interference pattern corresponds to a particular sheet thickness. By tracking the radial distance, *r*, from the collision point of a particular fringe (essentially a contour of constant sheet thickness, *h*) as a function of angle, the *φ* dependence of the product *hr* can be determined. By Eq. 1, this is proportional to *Q*(*φ*)/*u*_0_, which directly gives the angular distribution of *Q*(*φ*) if *u*_0_ is presumed independent of *φ* [which may (Bush and Hasha, 2004) or may not (Choo and Kang, 2002) be a well-founded assumption].

Measured curves for *hr* with respect to *φ* [i.e., *Q*(*φ*)], are shown in Figure 2B for the three nozzles. The curves shown are the average of several fringes on each sheet and represent the variation in thickness of the sheet at a constant radius from the collision point. The sheet thickness also depends on the chosen radius, *r*, per Eq. 1. For each nozzle style, the thickest part of the sheet was found to lie approximately along the centerline, with *φ* = 0°, with the sheet becoming progressively thinner with greater deviation from the centerline. The converging nozzle demonstrated the strongest *φ* dependence, with a 10% thickness change over just a ±10° deviation from the centerline. Surprisingly, the on-chip and off-chip colliding nozzles demonstrated very different profiles, where the on-chip colliding nozzle produced a sheet with a very small angular dependence (only ∼3% thickness change over ±10°), while the off-chip demonstrated greater variation and greater asymmetry in the flow (∼6% thickness change over ±10°). The notable asymmetry in the off-chip colliding sheet was likely a result of the polishing process producing slightly different apertures for the two jets. For all three nozzle types, the sheets tend to become flatter with distance from the nozzle. This flattening follows from the radial spreading of the sheet from the collision point described by Eq. 1.

Some characteristic sheet surface roughness can also be seen in Figure 2A, where bright and dark features can be seen with the interference pattern. These surface features arise from small-amplitude waves on the surface of the sheet which generally appear stationary under imaging. As can be seen from their minimal effect on the white light thin-film interference fringes in Figure 2A and has been previously examined with FTIR microscopy (Hoffman et al., 2022), these surface features cannot exceed tens of nanometers in height. That height is sufficiently large to affect the angle of incidence and result in bright and dark features on the sheet, but not large enough to affect the interference of visible light (wavelengths > 100 nm) and disrupt the observed fringe colors. While features on this scale can be significant for reflection measurements, they are unlikely to matter for most spectroscopic transmission measurements.

#### 3.1.3 Sheet thickness vs. flow rate

To examine the supposed independence of the sheet thickness on the liquid flow rate, the thin film interference patterns reflected from the sheets were again used (see *Materials and methods* section). A converging nozzle (*w*_0_ = 100 μm, *d* = 20 μm), an on-chip colliding nozzle (*R* = 25 μm), and an off-chip colliding nozzle (*R* = 25 μm, *d*_*c*_ = 700 *μ*m) were used in this comparison, as they gave sheet thicknesses which can be effectively monitored using the white light reflection method over a wide range of similar liquid flow rates.

**FIGURE 3:**
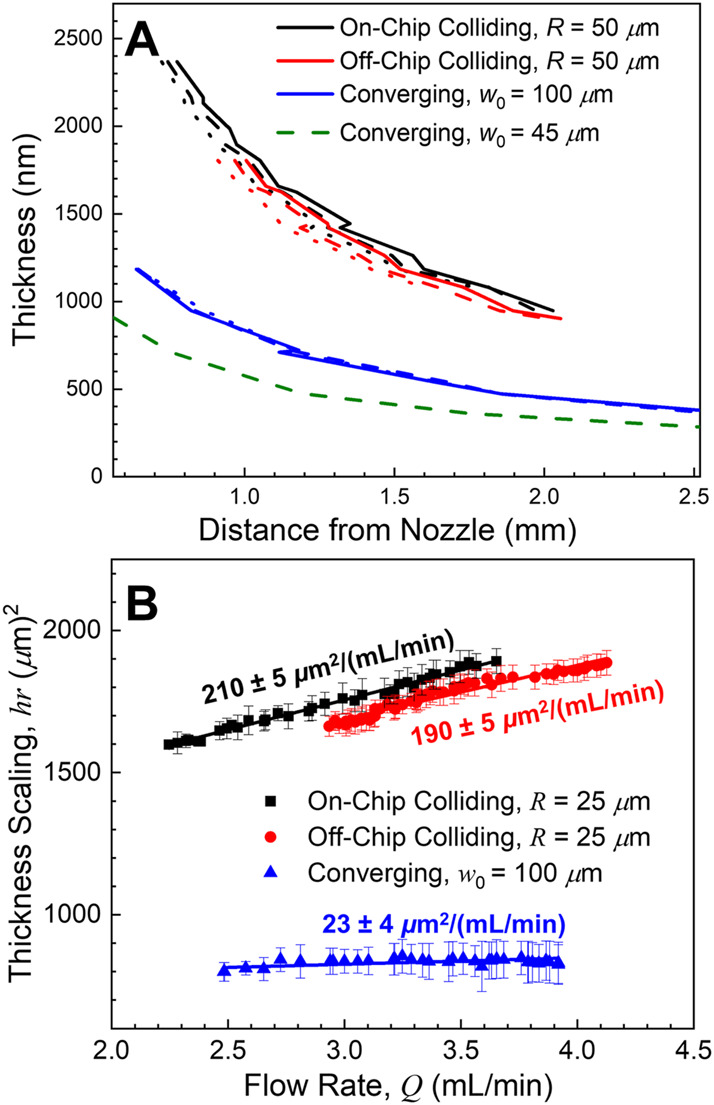
**(A)** Example sheet thicknesses along the centerline (*φ* = 0°) for the three nozzle types as a function of flow rate (*Q* = 3.0, 3.3, and 3.6 ml/min as dotted, dashed, and solid lines, respectively). The converging nozzle thickness profiles (blue curves) were essentially flow rate independent, while the colliding nozzle sheets became thicker with increasing flow rate (red and black curves). Green dashed curve shows the thickness profile for the thinnest sheet examined in this work (*Q* = 2.2 ml/min). **(B)** The sheet thickness scaling, *hr*, along the centerline as a function of flow rate. The two colliding nozzles exhibited almost the same linear rate of change in the thickness scaling with respect to flow rate, while the converging nozzle showed minimal changes.

Quantitative thickness profiles for the centerline of the sheets (*φ* = 0°) were extracted from the white light fringe patterns. Example profiles for the three examined nozzles are shown for three flow rates (Q = 3.0, 3.3 and 3.6 ml/min; dotted, dashed, and solid lines respectively) in Figure 3A. As expected, the converging nozzle profiles are essentially indistinguishable from each other (blue curves). Prior studies have found that the thicknesses of the sheets produced by colliding jets can have a detectable flow rate dependence (Choo and Kang, 2001), and here both colliding nozzles have a noticeable flow rate dependence, with the sheet generally becoming thicker with increasing flow rate (red and black curves). In all cases the assumption of constant speed for a given flow rate seems correct; *hr* is constant for a given flow rate (Eq. 1), but the value of *hr* varies with flow rate.

This thickness scaling (the conserved product of sheet thickness by distance from the collision point, *hr*) of the sheet as a function of flow rate was then determined for the three nozzles and fit with a linear regression (Figure 3B). Again, the off- and on-chip colliding nozzle sheet thicknesses (red and black points, respectively) increase with increasing flow rate, corresponding to a thickness increase of more than 10% over the examined flow rates. Both the thickness scaling and the flow rate dependence were nearly identical for the two colliding nozzles, despite the differences in the angular liquid flux shown in Figure 2. This high degree of similarity demonstrates that these properties are virtually unaffected by the location of the collision point relative to the nozzle.

Across the entire usable set of flow rates, the converging nozzle sheets (blue points) exhibited no change in thickness scaling within our ability to observe (corresponding to at most a 5% thickness variation across the full range of flow rates). The converging sheets’ thickness profiles appear to be effectively flow rate independent. The converging nozzles were also capable of producing the thinnest targets of the three styles and dimensions examined. The thinnest sheet examined as a part of this work was < 300 nm thick at its furthest point (Figure 3A, dashed green line. *w*_0_ = 45 μm, *d* = 20 μm, *Q* = 2.2 ml/min).

### 3.2 Sheet length and width

#### 3.2.1 Scaling

The extent of the liquid sheet’s expansion may be estimated by balancing the effect of inertia against that of surface tension:

$$h_{f}\rho u_{0}^{2}\propto \sigma$$ (3)

Here ρ and σ are the liquid’s density and surface tension, *u*_0_ is speed, and *h*_*f*_ is sheet thickness at the furthest distance from the nozzle. The left hand side of Eq. 3 is the outward force on a volume element due to the momentum of the fluid and the right hand side is the inward force due to surface tension. Using Eq. 2, this expression can be rewritten in terms of a maximum distance *r*, rather than a minimum thickness *h*_*f*_,:

$$r\left(h_{f}\right)\propto \frac{\rho u_{0}^{2}A}{\sigma }$$ (4)

A more formal derivation by Taylor (Taylor, 1960) found this maximum distance to be:

$$r_{T}=\frac{\rho Q^{2}}{2\sigma A}$$ (5)

where A is the cross-sectional area of the nozzle channels and $Q=Au_{0}$ . While strong deviations from the Taylor radius, *r*_*T*_, can be expected due to the influence of the sheet rims (Bush and Hasha, 2004), the length and width measurements for all of the nozzles examined in this work were found to be proportional to the Taylor radius.

#### 3.2.2 Sheet length

**FIGURE 4:**
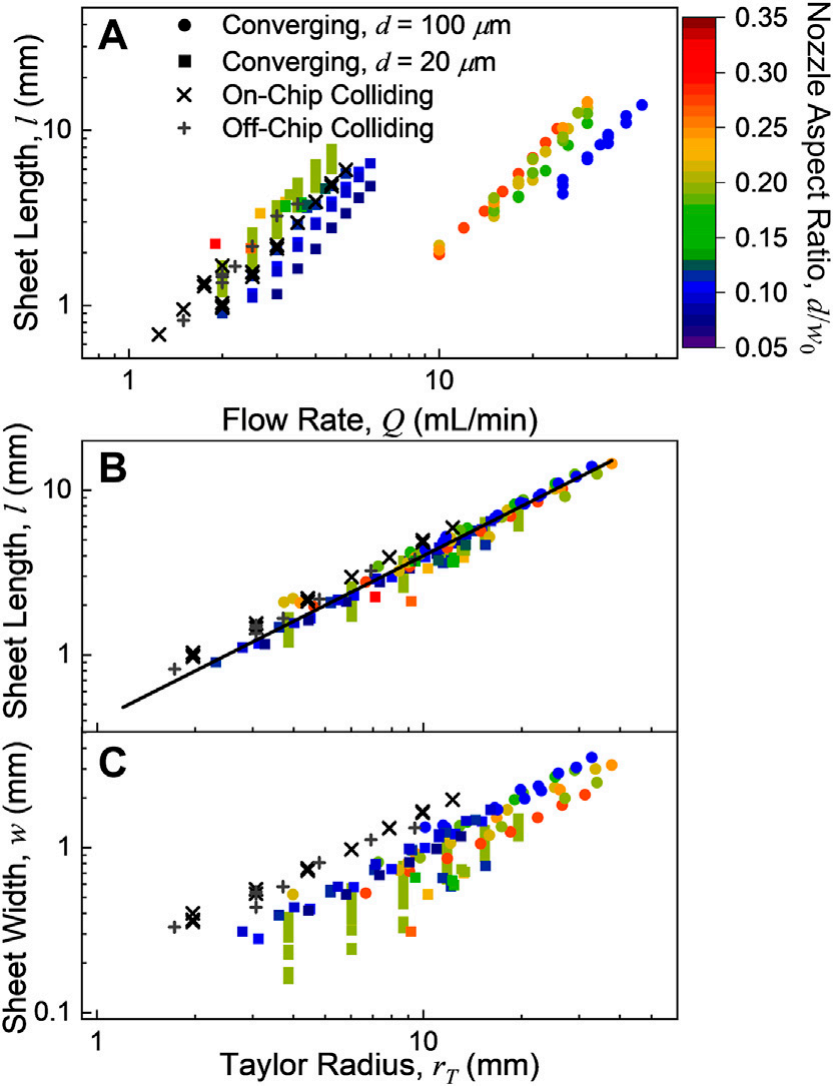
**(A)** Sheet lengths, *l*, as a function of flow rate, *Q*. **(B)** Sheet lengths as a function of the Taylor radius, *r*_*T*_ (Eq. 5), which is proportional to *Q* ^2^ and the hydrodynamic Weber number. All of the studied sheets had lengths *l ≈* 0.4 *r*_*T*_ (black line). **(C)** Sheet widths as a function of the Taylor radius. All of the sheet widths were proportional to the Taylor radius, but the colliding sheets were broader than the converging sheets. The width of the converging sheets decreased with increasing nozzle aspect ratio, *d*/*w*_0_.

The length of the liquid sheets (*l* in Figure 1A) as a function of flow rate for different converging nozzle exit widths and depths can be seen in Figure 4A in the units for which the measurements were made. Two nozzle depths were used (20 and 100 μm, squares and circles respectively) with multiple nozzle widths. The variation in area is expressed here in terms of aspect ratio (*d*/*w*_0_) of the nozzle exit. Well-formed sheets could typically be produced with lengths ranging from about 1 to 15 mm, depending on the nozzle channel dimensions. For a given nozzle, increasing the flow rate increases the length of the sheet quadratically (as predicted by the scaling in Eq. 5). Similarly, a larger area nozzle aperture (smaller aspect ratio for a given channel depth) requires a higher flow rate to reach the same sheet length.

The dimensions of the sheets produced by the colliding nozzles were found to have largely similar behavior to the converging nozzles. However, as the channel cross-sectional area of the colliding nozzles cannot be modified by polishing, only the two channel radii (20 and 25 μm) of on- and off-chip colliding nozzles could be readily examined. The sheet measurements for the on-chip and off-chip colliding nozzles are shown in Figure 4 as x’s and +’s, respectively. As can be seen in Figure 4, the differences between the on- and off-chip colliding nozzles are modest. These nozzles produced sheets with dimensions comparable to the *d* = 20 μm converging nozzles at similar flow rates (1–5 ml/min), with sheets ranging from 0.8 to 8 mm long and 0.2–2 mm wide.

In Figure 4B, the measured sheet length is compared to the Taylor radius from Eq. 5 using ρ = 0.997 g/cm^3^ and σ = 72.9 mN/m for water at room temperature (Speight, 2017). This scaling alone describes the variation in length with different nozzle aspect ratios, exit area, flow rate, and nozzle type. However, the measured lengths correspond to *l* ≈ 0.4 *r*_*T*_ (black line in Figure 4B), which is consistently smaller than the predicted length from the Taylor radius. This deviation likely relates to the effect of the sheet rims (Bush and Hasha, 2004), although it is notably consistent across a range of nozzle geometries.

#### 3.2.3 Sheet width

**FIGURE 5:**
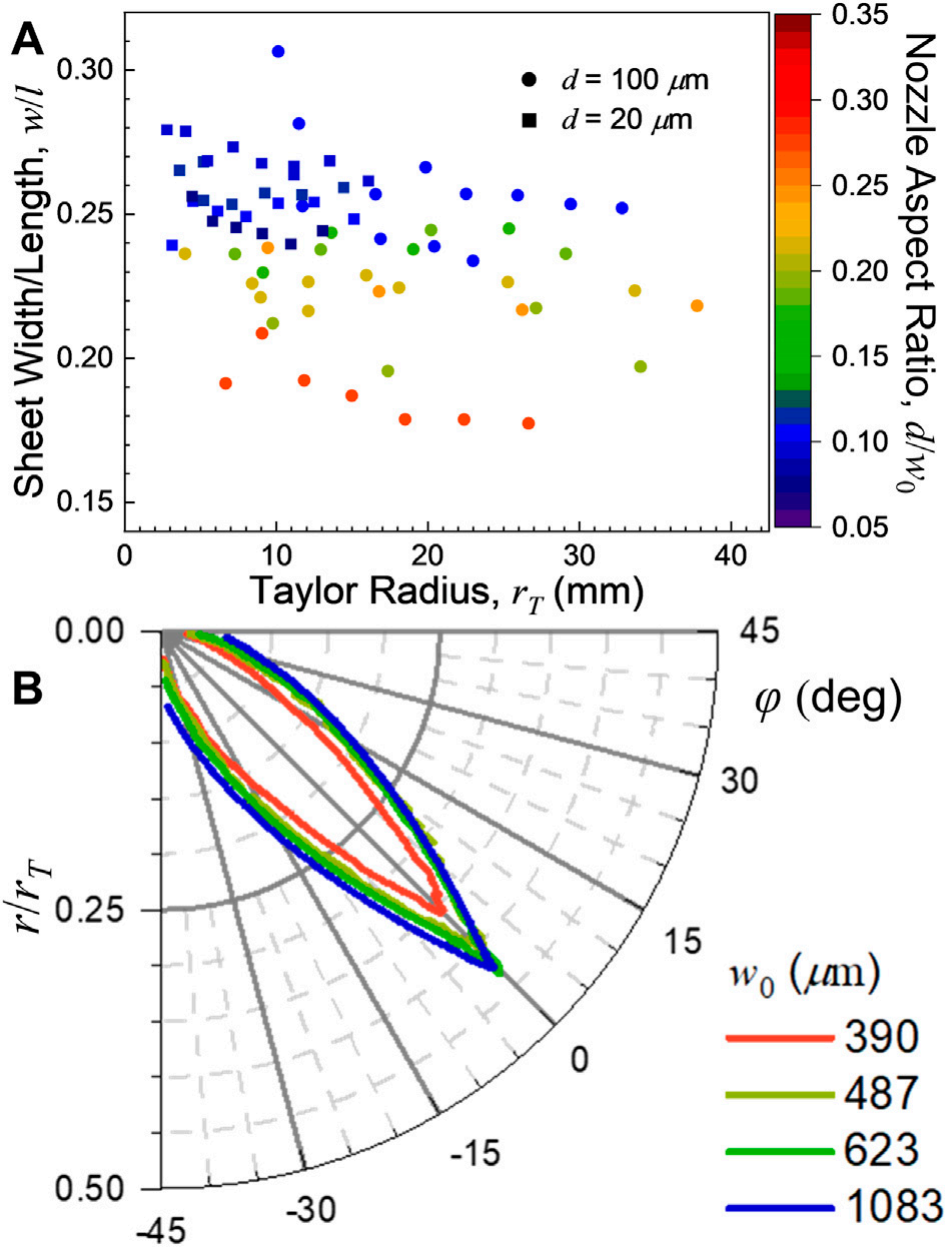
**(A)** Sheet width/length ratio (*w*/*l*) of the converging nozzle sheets as a function of Taylor radius, *r*_*T*_. The converging sheets with the largest aspect ratio (*d/w*_0_, most square nozzle exit) produced the narrowest sheets, but the width/length ratio did not strongly depend on flow rate. **(B)** Sheet profiles for several converging nozzle sheets (*d* = 100 μm) scaled to their Taylor radii. These scaled profiles were found to be virtually flow rate independent. The gradual narrowing of the sheet profile with increasing aspect ratio (decreasing *w*_0_) is apparent.

The sheet width (*w* in Figure 1A) vs. Taylor radius can be seen in Figure 4C. Sheet width has a much stronger dependence on the nozzle geometry through aspect ratio, nozzle type and converging/colliding angle than the sheet length. The colliding sheets were consistently wider than those produced by the converging nozzles. Amongst the converging nozzles, as the aspect ratio increases (i.e., the nozzle exit becomes more square), the sheet becomes somewhat narrower [as was found previously for polyimide converging nozzles (Ha et al., 2018)]. However, for all the nozzles studied, the sheet widths maintained the same quadratic dependence on the flow rate that the sheet lengths did. In other words, the ratio of the sheet’s width to its length remains essentially constant with flow rate. This effect can be seen in Figure 5A, where nozzles with similar aspect ratios have similar sheet dimension ratios at all flow rates rates (as *r*_*T*_ ∼ *Q* ^2^ per Eq. 5). However, nozzles with the largest aspect ratio examined produced sheets that were about 20% narrower than those with the smallest aspect ratio. The overall profile of the sheet itself was then found to be essentially independent of flow rate when scaled by the Taylor radius. Figure 5B shows the resulting profiles of various converging nozzles with different aspect ratios (all with *d* = 100 μm), demonstrating the minor variations in the sheet profiles with respect to *w*_0_.

**FIGURE 6:**
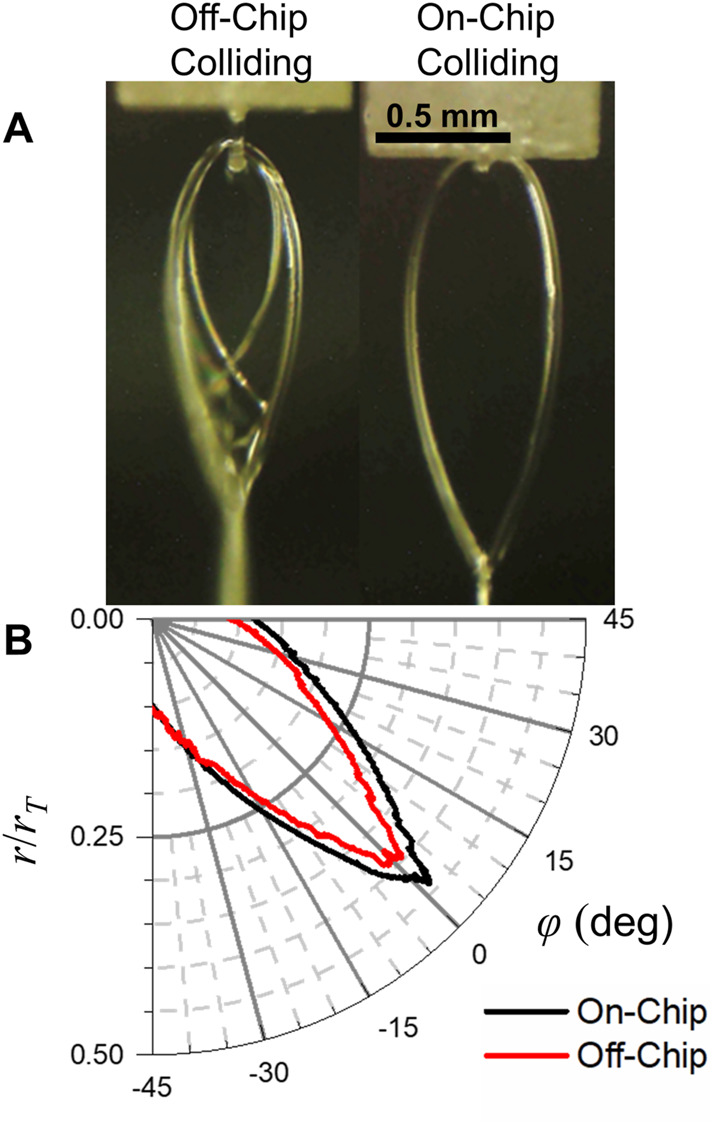
**(A)** Liquid sheets produced by an on- and off-chip colliding nozzle with the same channel radii (*R* = 20 μm) and the same flow rate (*Q* = 2 ml/min). The off-chip colliding sheet is somewhat smaller and has less stable cylindrical rims. **(B)** Sheet profiles scaled to the Taylor radii for the on- and off-chip colliding nozzles. The off-chip profile is smaller but otherwise similar to the off-chip profile.

While the differences between the on-chip and off-chip colliding nozzles were found to be relatively small, there were some consistent differences between the two. In general, it was found that the off-chip colliding sheets were slightly smaller than equivalent sheets produced with the on-chip colliding nozzles. An example of sheets produced with on- and off-chip colliding nozzles (*d*_*c*_ = 510 μm for the off-chip colliding nozzle, *R* = 20 μm, *Q* = 2 ml/min for both nozzles) are shown in Figure 6A and their scaled profiles are shown in Figure 6B. Additionally, the rims of the off-chip colliding sheets were frequently unstable, and appeared more like a spray than a well-defined cylindrical rim seen in the on-chip colliding and converging nozzles (e.g., Figures 2A, 6A). As was seen in Figure 2A, the off-chip colliding nozzles were still capable of producing flat, smooth targets despite the unstable rims.

### 3.3 Vacuum performance and recirculation

**FIGURE 7:**
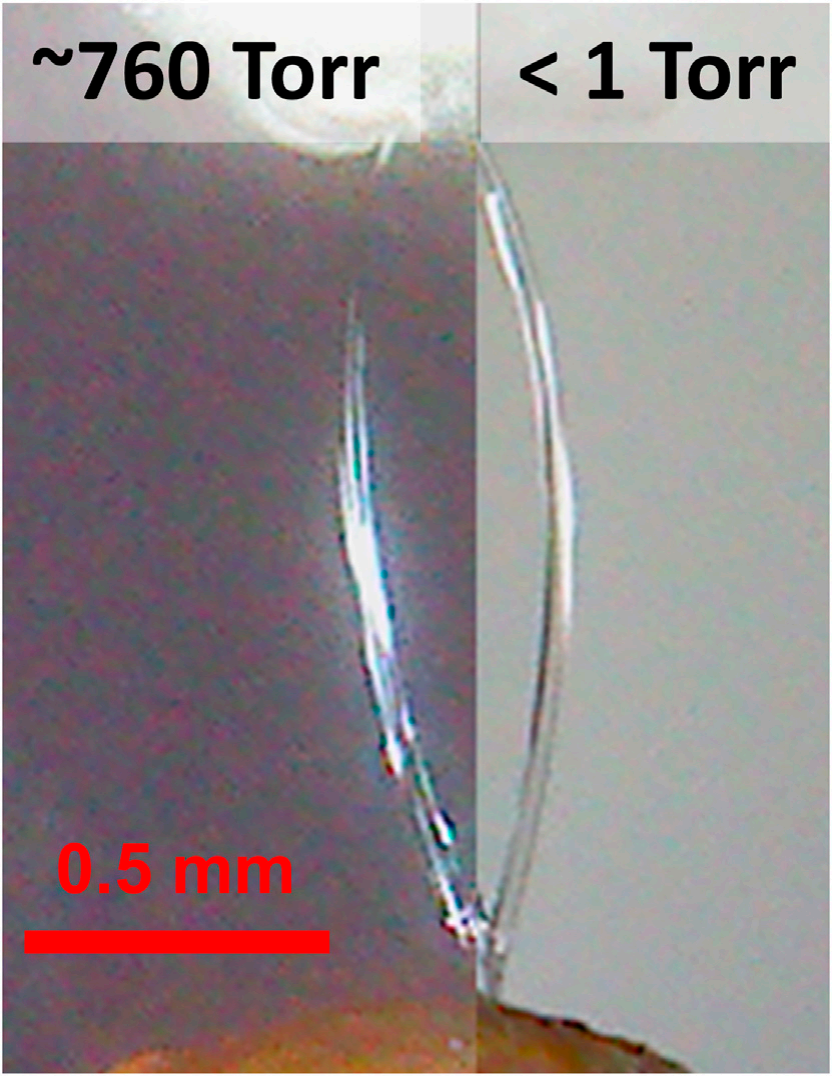
A converging nozzle (*d* = 20 μm, *w*_0_ = 100 μm) running in atmosphere (left) and in vacuum (right) at a flow rate of *Q* = 2 ml/min. The sheet has the same profile in both cases.

The stability of the liquid sheets in vacuum has been critical for their use for optical and particle beams with extremely small attenuation lengths. Tests in vacuum showed no change in the profile of the sheet jet when compared to its operation in air, which demonstrates that the dimensional scalings shown in the previous sections can be directly applied to vacuum operation as well. This behavior is shown in Figure 7, which demonstrates the conserved sheet profile produced by a converging nozzle (*d* = 20 μm, *w*_0_ = 100 μm, Q = 2 ml/min) in air and vacuum. Prior work has also shown that the thickness profile of these sheets is similarly conserved in vacuum to within experimental error (Ekimova et al., 2015; Galinis et al., 2017).

**FIGURE 8:**
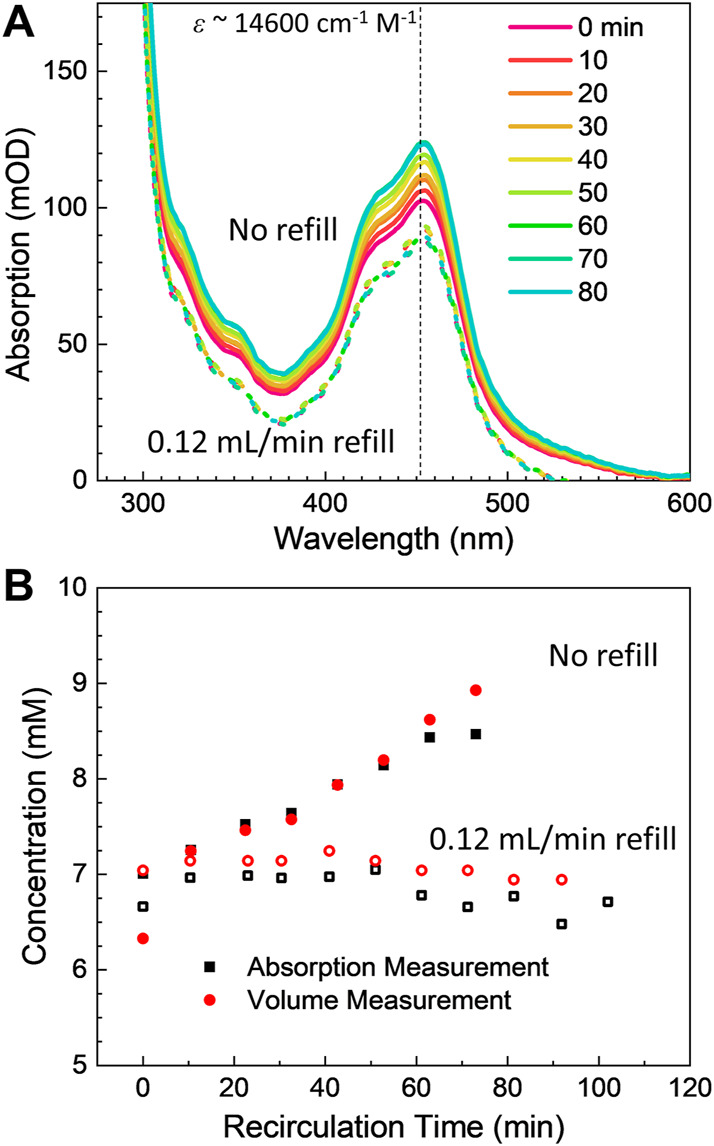
**(A)** UV-Vis absorption spectra of the test Rubipy solution during in-vacuum sample recirculation tests. Over time, the concentration of the Rubipy solution was seen to increase due to evaporative losses in vacuum (solid curves). When the sample reservoir was refilled at a 0.12 ml/min flow rate, the sample solution concentration stabilized (dotted curves, vertically offset for clarity). **(B)** Recirculated Rubipy sample concentration over time with and without refilling the reservoir for evaporative losses (filled and open symbols, respectively). The black points are the concentration as determined from the UV-Vis absorption spectra, while the red points were extrapolated from the reservoir volume assuming only water was lost during the recirculation process.

Sample recirculation is frequently required for running small sample volumes for extended periods of time. However, running microjets in vacuum also results in rapid solvent evaporation which can inadvertently concentrate the solute. To test the effects of solvent evaporation on the operation of the recirculation system, a Rubipy solution was run in vacuum using a converging nozzle (*w*_0_ = 216 μm, *d* = 20 μm) at a flow rate of 3.5 ml/min, suitable for producing a several *μ*m thick target. The concentration of the Rubipy sample was then monitored using UV-Vis spectroscopy (see *Materials and methods*) and the volume of the sample reservoir was monitored over time as the sample solution was run in vacuum. As the sample ran in the vacuum chamber, the concentration of the sample noticeably increased over the span of just minutes. Example UV-Vis absorption spectra can be seen in Figure 8A (solid curves), where the intensity of the Rubipy absorption clearly grows over time, indicating a more concentrated solution. The concentration of the sample over time is shown in Figure 8B (filled black points), which shows that the sample concentration increased by nearly 20% over the 80-min test.

The sample concentration increase was tightly correlated with the total volumetric loss in the sample reservoir over the same duration (Figure 8B, filled red points). The sample reservoir total volume appeared to lose slightly more than 0.1 ml/min over the span of the test, corresponding to an approximately 8 ml total drop from an initial starting volume of 40 ml. This volumetric drop neatly corresponds to the 20% sample concentration increase which was seen over the same time period, which demonstrates that the volumetric loss arises from the evaporative loss of volatile water while the essentially nonvolatile Rubipy salt was concentrated in the remaining solution. To confirm this observation, the same experiment was repeated with a second HPLC pump delivering on average 0.12 ml/min of water to the sample reservoir. This refill procedure stabilized both the concentration of the solution and the volume of the reservoir (Figure 8A, dotted curves and Figure 8B, open symbols).

### 3.4 Sheet damage from intense X-ray free electron lasers pulses

Converging sheet nozzles with thickness of order ∼20 μm (*w*_0_ = 578 μm, *d* = 100 μm) are well suited for ultrafast hard X-ray pump-probe measurements. As the group velocity mismatch in water is on the order of 1.1 fs/μm and the typical X-ray and laser durations at an XFEL are 40 fs, the sheet thickness no longer contributes negatively to a loss of time resolution. Since the X-ray signals scale with the thickness of the jet, it is also not advantageous to use thinner jets than necessary. High intensity pulses from XFELs have been previously shown to induce explosions’ in cylindrical liquid microjets (Stan et al., 2016). Here we did preliminary investigations of such explosions in liquid sheets.

**FIGURE 9:**
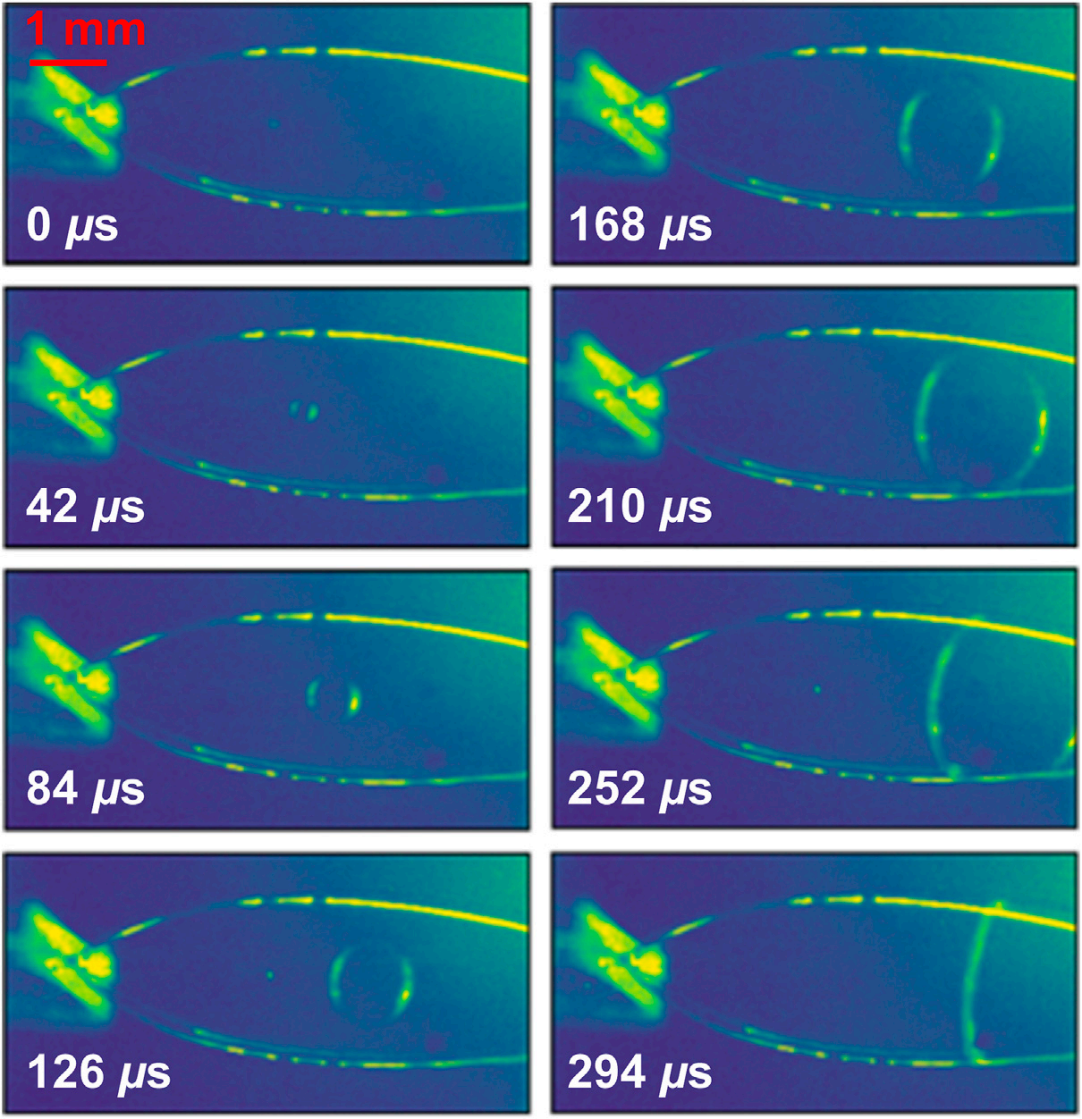
Single shot pictures of damage to a liquid sheet at a given delay following an intense XFEL pulse at LCLS. The sheet was produced by a converging nozzle with *d* = 20 μm and *w*_0_ = 578 μm. The resulting sample explosion causes a bubble that expands and flows downstream following the X-ray pulse.

When the intense XFEL pulse was tightly focused on the liquid sheet (spot size ∼5 μm, ∼0.5 mj, 9.5 keV, 40 fs duration), it was found that the high intensity pulse was able to punch a “hole” in the liquid sheet. In Figure 9, the expanding hole in the sheet jet can be seen moving downstream at the flow speed of the jet (*u*_0_ ∼ 10 m/s). Simultaneously, the hole radius expands at a constant speed of ∼4 m/s. By contrast, more defocused XFEL pulses (spot sizes of ∼30 and ∼130 μm) did not show the visible hole punching caused by the intense pulses.

## 4 Discussion

### 4.1 Sheet size

Ideally, the large areas of sheet jets can allow intense incident beams to be defocused while still hitting the target sample, which helps avoid sample or structural damage without sacrificing any linear spectroscopic or scattering signals. Towards this end, we consider suitability as a sample target based on the target’s dimensions and thickness variation over a reasonable range of the incident beam’s spot size.

Selecting an appropriate target requires selecting the appropriate nozzle geometry such that the sheet is larger than the beam’s spot size and the range in thickness over the probe dimensions is acceptable. In all cases, the maximum accessible sheet width and length was on millimeter scale. Both the sheet lengths and widths varied directly with the Taylor radius, which itself scales quadratically with the flow rate. This implies that the total surface area of the sheet then scales with *Q* ^4^. Small increases in the liquid flow rate can therefore dramatically increase the available target area, even though stable sheets can only be produced with a relatively narrow range of flow rates. At any flow rate within the stable range for a given nozzle, the sheet will have the same shape (e.g., length to width ratio), although the shape can be changed with colliding/converging angle *θ* or (in the case of converging nozzles) nozzle exit aspect ratio. The variation in thickness across the sheet then has to be considered when characterizing the optical path length. These thickness variations are most significant near the collision point, as the range of *φ* sampled in a spectroscopic experiment with a given spot size increases with decreasing radial distance, *r*, from the collision point.

For both the colliding (Bush and Hasha, 2004) and the converging nozzles (Ha et al., 2018), an increase in the colliding/converging angle *θ* (Figure 1B) is known to increase the width of the produced sheet. As the colliding angle examined here (40°) is substantially smaller than the converging angle (60°) and the colliding sheets were still found to be wider than the converging sheets, colliding nozzles should generally produce wider sheets than converging nozzles with comparable values of *θ*. A broader angular distribution of the fluid flow should result in thinner sheets if fluid speed is similar, per Eq. 1. However, this observation does not necessarily mean that colliding nozzles can produce thinner targets; for any given nozzle a higher flow rate produces a longer sheet which makes a thinner part of the sheet accessible. Compared with the colliding nozzles, the converging nozzles tested here were able to operate stably over a wider range of flow rates, producing sheets approximately twice as long as those produced by comparable colliding nozzles (Figure 4). The stable operation at high flow rate exhibited by the converging nozzles allows access to extremely thin parts of the sheet. For example, a 250 nm target for a 200 µm diameter electron beam probe has been produced using the converging nozzles described here (*d* = 20 μm, *w*_0_ = 60 µm) (Crissman et al., 2022).

As was shown by Ha et al. (2018) for converging nozzles, sheet thickness at a given distance from the nozzle is determined only by the nozzle’s geometry. Length scale invariance implies that larger nozzles produce thicker sheets for the same distance from the collision point. In particular, Eq. 2 shows that the converging nozzles can be modified to produce thicker sheets after manufacture through polishing, as the polishing procedure increases the width of the nozzle exit (*w*_0_). These modifications provide an additional way of tailoring the batch-produced nozzles to a given experiment. The size of the X-ray focus is generally kept within a prespecified range, so the choice of nozzle for any experiment can usually be determined beforehand to optimize for thickness and thickness variation across the XFEL spot.

In contrast to this work, the dimensions of the sheets produced by the converging nozzles described in Ha et al. (2018) were found to more strongly vary with nozzle aspect ratio (*d*/*w*_0_). The converging nozzles made by Ha et al. (2018) used a polyimide spacer to define the channel, and had a rectangular nozzle exit as opposed to the rounded rectangular exit produced by the isotropic etch for the nozzles examined here and illustrated in Figure 1C. These differences between nozzle designs may have also influenced the observed sheet thicknesses, with the polyimide nozzles having an aspect ratio dependence not found in the isotropically etched nozzles (Crissman et al., 2022).

### 4.2 Stability

On- and off-chip colliding jet nozzles showed similar behavior in their physical dimensions and flow rate dependence, but the greater stability provided by the on-chip colliding nozzle suggests that it is the superior nozzle design for most spectroscopic applications. The off-chip colliding nozzles described in this work require no post-production alignment; the channels are lithographically defined. However, the inability to make alignment adjustments is at times also a liability. The angle that the liquid jets leave the nozzle surface is dependent on the surface quality, which may change over time due to sample deposition. This buildup could cause the sheet center to drift or lead to nozzle failure. On-chip collision is far less sensitive to such changes but can be affected by nozzle damage as noted in Section 4.5.

Converging nozzles were generally stable over a larger range of flow rates. As mentioned previously, the greater range in flow rate for converging nozzles may also allow access to comparably thinner sheets. Colliding nozzles may also require adjustment to individual channel flow rates to maintain stability, a problem that does not occur for single-channel converging nozzles. The colliding nozzles used in this work had a single channel that split on-chip leaving no possibility to adjust the individual flow rates. The relative merits of using decoupled channels were not investigated in this work.

For all nozzle types, independence of the converging and colliding sheet thickness on the liquid flow rate has great benefits for the use of sheet jets in spectroscopic experiments. If the thickness of the sheet depends on the flow rate, then an unstable flow rate can induce thickness variations that can contribute noise to a spectroscopic measurement. The additional stability in the sheets’ thicknesses is a substantial benefit of the converging nozzles over the colliding nozzles (Figure 3), especially in cases where the stability of the flow rate cannot be ensured.

### 4.3 Vacuum operation

The behavior of the sheet jet in vacuum is dominated by the dramatic evaporation experienced by the sheet while it is exposed to the vacuum. Most directly, the vapor immediately increases the ambient pressure in the vacuum chamber (pressures of 10^–3^ Torr are typical even with robust 2.7 kl/s turbo pumping), requiring substantial differential pumping for many measurements. The sheet jet also undergoes rapid evaporative cooling. Previous studies of microjets in vacuum using Raman thermometry (Wilson et al., 2004; Chang et al., 2022) and electron diffraction measurements (Nunes et al., 2020) have found dramatic cooling rates on the order of 10^5^–10^6^ K/s. This extreme rate of cooling means that different distances from the nozzle will exhibit noticeably different temperatures (typically varying 10 s of K across the sheet), which could have significant effects on the solubility of dissolved compounds, and could complicate [or enable (Sellberg et al., 2014; Laksmono et al., 2015)] measurements of temperature-sensitive properties such as chemical dynamics or kinetics. It has been shown previously (Nunes et al., 2020) that proximity to the heated catcher can increase the fluid temperature at the nozzle exit, although with much lower fluid flow (200 μl/min) than was used here. Aside from cooling and evaporative loss, the converging sheet nozzles appear to operate the same in vacuum or ambient air—sheet dimensions were not affected.

For expensive or difficult to synthesize samples, it is generally desirable to reuse a small volume of sample repeatedly throughout an experiment, especially with the relatively high flow rates required to operate many of the sheet nozzles examined in this work. While this is straightforward to achieve in atmosphere, recirculating a sample in vacuum is a more challenging task. Towards this end, a recirculation system was developed (see Methods section) to reuse the same sample volume repeatedly as the jet ran in vacuum. A sample recirculation scheme was detailed for running sheets in vacuum, which allowed for a 40 ml volume to be used at a 3.5 ml/min flow rate for over 3 h, the duration of this test. At this flow rate, the entire sample volume would have been consumed in just over 10 min without recirculation. This recirculation scheme is critical for the operation of these thicker sheet jets in vacuum—especially with precious samples—which is necessary for extreme ultraviolet and soft X-ray spectroscopies. With constant replenishment of the solvent lost to evaporation, sample concentration may be held constant indefinitely. Additional care would need to be taken for solutions containing multiple volatile components.

The high flow rates (1–50 ml/min depending on nozzle choice and desired sheet dimensions) required for the operation of these nozzles still necessitates a substantial sample volume even with recirculation. While the sample is not consumed, the swept volume of the entire recirculation system was approximately 10 ml (primarily arising from the pressure fluctuation dampener). The swept volume then provides the minimal amount of sample required to run the nozzle. Additionally, the sample must also be able to survive repeated recirculation. These features make these nozzles better suited for solution-phase scattering and spectroscopy experiments as opposed to serial femtosecond crystallography or related methods which deal with delicate biological samples.

### 4.4 Samples and clogging

While this work primarily examined neat water, many of these results should be readily applicable to liquid systems with different viscosities, surface tensions, and densities. Previous work on colliding (Bush and Hasha, 2004) and converging (Ha et al., 2018) nozzles have found that sheet dimensions depend negligibly upon the liquid viscosity. Bush and Hasha found that higher viscosity fluids tended to produce more stable sheet structures. However, the pressure required to supply a given flow rate increases roughly linearly with viscosity, so there is a practical limit to the viscosity of a sample which can be used based on the strength of the etched glass nozzle. The nozzles examined here tended to fail between 2,000–3,000 psi, with normal operating conditions for water requiring several 100s of psi. This gives a practical maximum viscosity of ∼10 cP for these nozzles.

Surface tension and liquid density enter directly into the Taylor radius calculation in Eq. 5. To first order, the sheet length/width will increase proportionately with density and inverse-proportionately to surface tension. The sheet thickness scaling is known to be only weakly impacted by these properties. As a result, a lower surface tension liquid (e.g., isopropanol) can produce sheets more than twice as long as water for the same flow rate and allow access to thinner parts of the sheet.

Hard X-ray probes require thicker sheets (∼20 μm) than soft X-ray probes (∼5 μm). The largest of the converging nozzles used in this study produce sheets of tens of microns thickness. Clogging becomes problematic for circular nozzles under 20 μm, which suggests that colliding and converging nozzles with nozzle dimensions larger than 20 μm are useful for producing sheet thicknesses of 20 µm or less. For thicker sheets, “waterfall” type nozzles with a single straight rectangular aperture in which the sheet dimensions are similar to the nozzle aperture size should suffice.

Converging nozzles have shown additional benefits with regard to clogging. The probability of a given nozzle clogging is greatly reduced with increasing aperture size. A single channel converging nozzle with similar exit aperture area to the combined area of the two channels in a colliding nozzle will be less susceptible to clogging for similar thickness sheets. While only solutions of small organics and salts analogous to the Rubipy solution discussed above have been tested, no converging nozzle has unambiguously failed in operation due to sample-induced clogging. Salt build-up on the nozzle or catcher in-vacuum was occasionally problematic but could be washed off by briefly switching from sample to neat water.

### 4.5 Damage

Liquid samples may be damaged by interaction with X-rays on various timescales. On very short timescales, irreversible intrapulse changes driven by ionization have been shown to occur at high intensity, 10^17^ W/cm^2^ (Alonso-Mori et al., 2020). On somewhat longer timescales but similar intensity, mechanical damage driven by energy transfer to solution may cause heating, compression [as clearly seen with time-resolved solution scattering at (Kjær et al., 2013)], or other mechanical damage [as is common in high intensity applications such as serial crystallography (Grünbein et al., 2021)]. On longer timescales damage may arise from oxidation, dehydration and heating.

Figure 9 shows an example of mechanical damage—a hole punched into a liquid sheet by an intense XFEL pulse. Fortunately, the supercritical flow in the liquid sheet (Bush and Hasha, 2004) ensures that the produced hole is carried downstream by the jet (∼10 m/s) faster than it can grow (∼4 m/s). The liquid target is then not catastrophically disrupted by the hole formation, and it reforms stably after the hole flows out of the sheet. In addition to the visible hole in the jet, a sonic pressure wave is also launched upon impact. This density wave is not visible in the images and propagates at the speed of sound in the liquid. This pressure wave provides the main challenge for higher repetition rate sample delivery, as it is not possible to flow these jets supersonically, which would be necessary to outrun the expanding pressure wave. The suppression of this hole punching effect and the reduction in amplitude of density waves by increasing the beam spot size also demonstrates the use case for sheet jets as large-area targets for XFELs.

Increasing the target size does not necessarily escape all damage types, especially on the longest timescales, even though a decreased photon density generally reduces photodamage effects. It is known from many synchrotron studies that X-ray induced photochemistry occurs in samples, of which biological samples seem to be the most vulnerable. Two main effects are usually observed: photoreduction or an impact on the active site structure (Penner-Hahn et al., 1989; George et al., 1999; Ascone et al., 2003; Levina et al., 2005; Yano et al., 2005; Corbett et al., 2007; Antonyuk and Hough, 2011). Even though in some cases photo-oxidation has been found, it seems to be mostly limited to lower photon energies such as the sulfur K-edge (Hackett et al., 2012). Solutions of biological molecules in X-ray experiments are often dilute, and to a first approximation the radiochemical properties of water will be the most important factor in solution. Photolysis in samples containing water is relatively well understood. Water decomposes under ionizing radiation into a handful of major species, namely hydrated electrons (e^−^aq), hydroxyl radicals (OH*), the water radical cation (H_2_O^+^), hydrogen radicals (H*), hydroxonium ions (H_3_O^+^) and hydrogen peroxide (H_2_O_2_) (Gunter, 1967; Plonka et al., 2000; Garrett et al., 2005; El Omar et al., 2011). Among these products, solvated electrons are likely most relevant to photoreduction (Frank et al., 2019; Stellato et al., 2019). Because the nozzle exit aperture is much larger than the sheet thickness, samples can run at high concentration with minimal risk of clogging. Using larger aperture nozzles to produce thin sheets is an excellent way to produce a target at high concentration with an attenuation length still suitable for soft X-ray studies. Sheet thickness is independent of the thermophysical properties of the fluid, so addition of scavengers is possible without change to thickness. Sulfate and nitrate can potentially give some protection from photoreduction by scavenging hydrated electrons, as found in liquid solution (Mesu et al., 2006; George et al., 2012). Sodium ascorbate has also been used as a scavenger in an Fe(III)-myoglobin sample (Hersleth and Andersson, 2011). The increase in total absorbed dose, however, has only been small or moderate (Beitlich et al., 2007; Macedo et al., 2009). The usable lifetime of a given volume of sample might eventually be limited to long timescale degradation regardless of sample delivery method.

Also on long timescales, nozzles may fail due to accumulated damage to the nozzle from high intensity beams. We have observed failure of the on-chip colliding nozzles at X-ray intensities of order 100 µJ focused into a 100 µm spot when the interaction point is relatively close (100 µm) to the chip. This is consistent with what we have also observed for gas-accelerated sheets described elsewhere (Koralek et al., 2018) when using optical or X-ray sources. In both cases no damage to the nozzle was observed. Larger converging nozzles have operated at much higher intensity without similar failure. We have also observed changes to the hydrophobic coating of other types of nozzles (drop dispensing) when operated near a high intensity beam. It may be that that the hydrophobic coating of the nozzles described in the manuscript are similarly affected, as the gas accelerated and the on-chip colliding nozzles both rely on a hydrophobic coating for their operation, while the converging nozzles do not.

## 5 Conclusion

Microfluidic liquid sheets are promising large-area, solution-phase targets for current and next-generations of high repetition rate XFELs. The key dimensions of sheet jets produced with several styles of isotropically etched glass nozzles were examined as a function of flow rate, along with the behavior of the jets in vacuum. Both the colliding and converging nozzles examined here exhibited the characteristic 1/*r* thickness scaling that was (almost) flow rate independent and sheet lengths and widths which scaled quadratically with the flow rate. It was found that the sheets produced by the colliding nozzles were wider and flatter than those produced by the converging nozzle, but the colliding nozzle sheet thicknesses had a measurable flow rate dependence. A recirculation system for in-vacuum operation was also demonstrated, which is vital for soft X-ray spectroscopy of small sample quantities.

The effects of damage to the sheet structure, sample, and nozzle itself from high intensity XFEL pulses remain a concern, especially with next generation, high repetition rate light sources coming online. The ability of intense XFEL pulses to punch holes in the liquid sheets was demonstrated, as well as the fluid dynamic properties of the sheet which ensures that the punched hole is always downstream of the incident X-ray beam. Critically, it was found that defocusing the beam was sufficient to stop the structural damage to the sheet while keeping the entire beam incident on the sheet. Ideally, a similar strategy can be employed to minimize radiative damage to samples and microfluidic nozzles as well.

## Data Availability

The raw data supporting the conclusion of this article will be made available by the authors, without undue reservation.
