# Supplementary material for: Microfluidic liquid sheets as large-area targets for high repetition XFELs
Source: Front Mol Biosci. 2022 Dec 9;9:1048932. doi: 10.3389/fmolb.2022.1048932 (PMC9780453; doi:10.3389/fmolb.2022.1048932)
Supplement: Supplementary file 1 [file DataSheet1.PDF]

Supplementary Material

1 Sheet Width and Flow Rate

Several additional converging nozzle types were tested while exploring the process for glass fabrication. The nozzle exit shapes varied sufficiently to prevent a simple expression for Taylor radius, however the liquid sheet shape dependence on flow rate, *Q*, seemed consistent for all nozzles. Liquid sheet width-to-length ratios were measured for 23 nozzles of 14 different shapes. In all cases the ratio of sheet width-to-length was flow rate independent if the sheet was measured from just inside the sheet rims at the widest part of the sheet. The rims of the sheets remained roughly the same diameter over the flow rates observed, 1 mL/min to 4.5 mL/min. Measuring sheet width from the outside of the sheet rims then effectively adds a constant to the width as measured from just inside the rims. Assuming both the inner measurement of width and the length vary with Q^2, the rim thickness contributes fractionally less for larger sheets at higher flow rate. This effect causes the mild dependence on *Q* for width/length ratios as measured from the outer edges of the sheet seen here in supplementary figure 1, and possibly the work of Ha et al., which measured sheet widths from the outer edges.

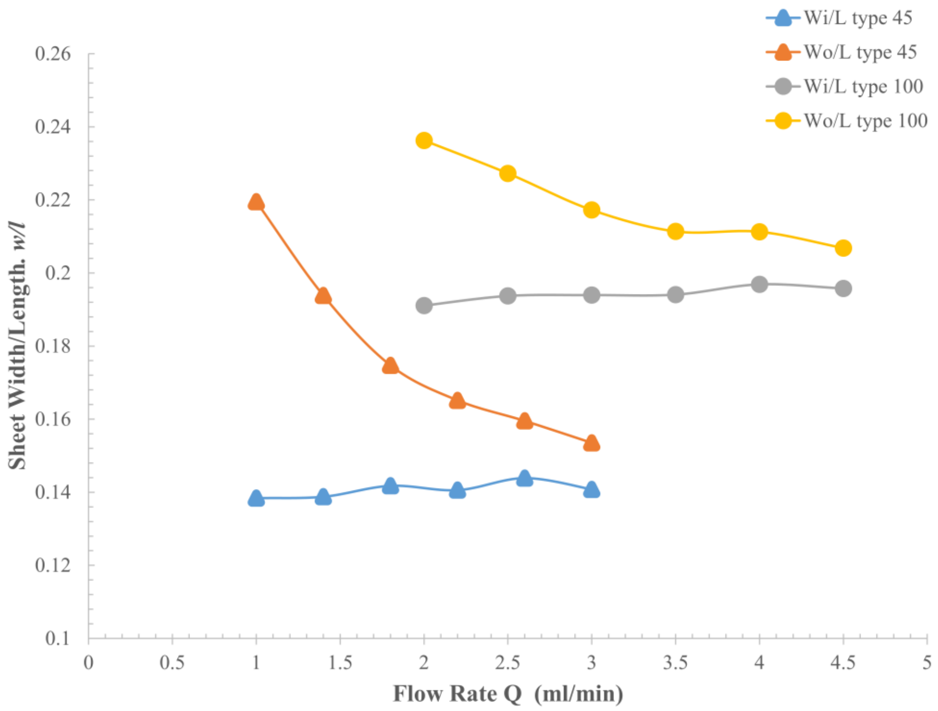

Supplementary Figure 1. Sheet width to length ratio as measured from the inside of the sheet rims, *Wi* is compared to data measured from to outer edges of the rims, *Wo*. The exit aperture of all nozzle apertures has depth 20 μm. Nozzle exit width is approximately 45 μm for type 45 nozzles and is averaged over 8 different shapes and 10 nozzles total. Nozzle exit width is approximately 100 μm for type 100 and averaged over 6 shapes and 13 nozzles total.

2 Sheet Thickness and Nozzle Type

Colliding jet nozzles have a wider angular flow distribution, $Q(\theta)$, than converging nozzles. By Eq. 1, colliding jet nozzles then produce thinner sheets than for comparable size converging nozzles at similar flow rate. In supplementary figure 2, sheet thickness for colliding and converging nozzles are compared. Data for a colliding nozzle used in this study and colliding jet sheet thickness data published elsewhere by Ekimova et al., are shown. Ideally converging nozzles of identical convergence angle and exit area to that of the colliding nozzles would be used for comparison but such data was not available. Instead, the model of Ha et al., which agrees well with other data we have collected for converging nozzles, was used. Models also exist for colliding nozzles but have been typically compared to much larger sheets. Here, we have chosen to compare experimental colliding data to the predictions of the converging model.

Ekimova et al., used colliding jets with diameters of 45 μm and a colliding angle of 22.5 degrees. That data is compared to the curve labeled Ha 1 which was generated for a nozzle of exit dimensions depth 20 μm, width 160 μm and converging angle of 22.5 degrees. The converging nozzle dimensions were chosen to give the same total exit area as the colliding nozzle. The nozzle depth 20 μm was chosen as the lower limit to avoid clogging. Similarly, the nozzle used in the main text, here labeled as “SLAC”, with channel diameters of 50 μm and converging angle of 40 degrees was compared to a converging nozzle with exit depth 20 μm and width 196 μm and converging angle 40 degrees.

In both cases the converging nozzles produced thicker sheets, as would be expected due to the larger angular distribution of flow. In both cases, the colliding sheet was approximately half as thick as predicted by the converging nozzle model. The SLAC and Ekimova nozzles had similar dimensions and produced similar sheets. The larger converging angle in the SLAC nozzle produced the thinner sheet for the same distance from the colliding point. However, the larger angle nozzle was only useful for a narrower range of flow rate, 1 mL/min to 2 mL/min, while the smaller converging angle produced a stable sheet from 0.5 mL/min to 3 mL/min.

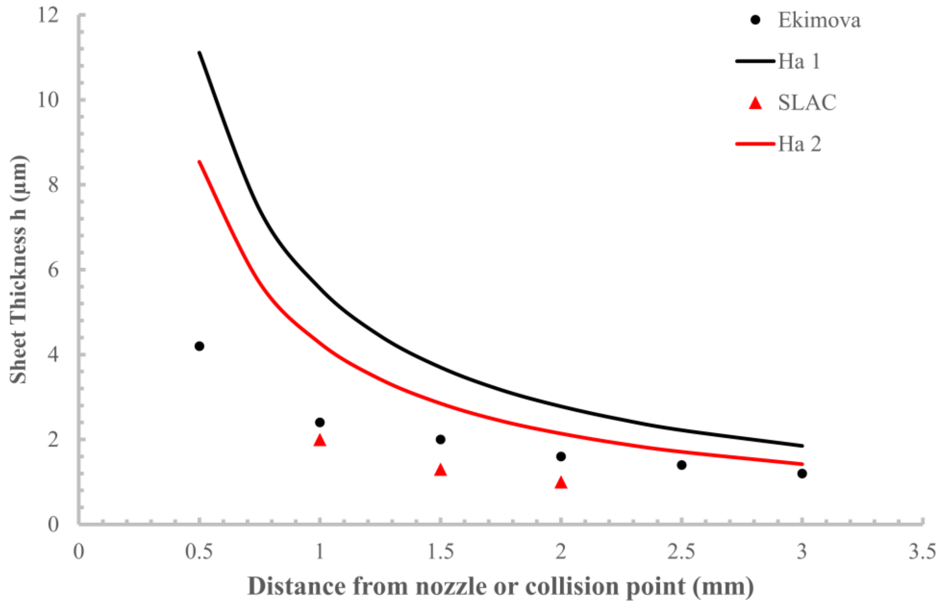

Supplementary Figure 2. Comparison of data for colliding nozzles symbols with model for converging nozzles, solid lines. Circles are data published by Ekimova for 45 μm diameter colliding nozzles at a 22.5° converging angle. Triangles are the colliding data presented in the main text for a 50 μm diameter colliding nozzle at a 40° converging angle. For comparison to converging nozzles, the Ha model with exit area and converging the same as colliding nozzles was used. A nozzle exit depth d = 20 μm was chosen to obtain an aspect ratio similar to those examined here and in Ha et al. The curve marked Ha 1 compares to the Ekimova nozzle and Ha 2 compares to the SLAC nozzle.

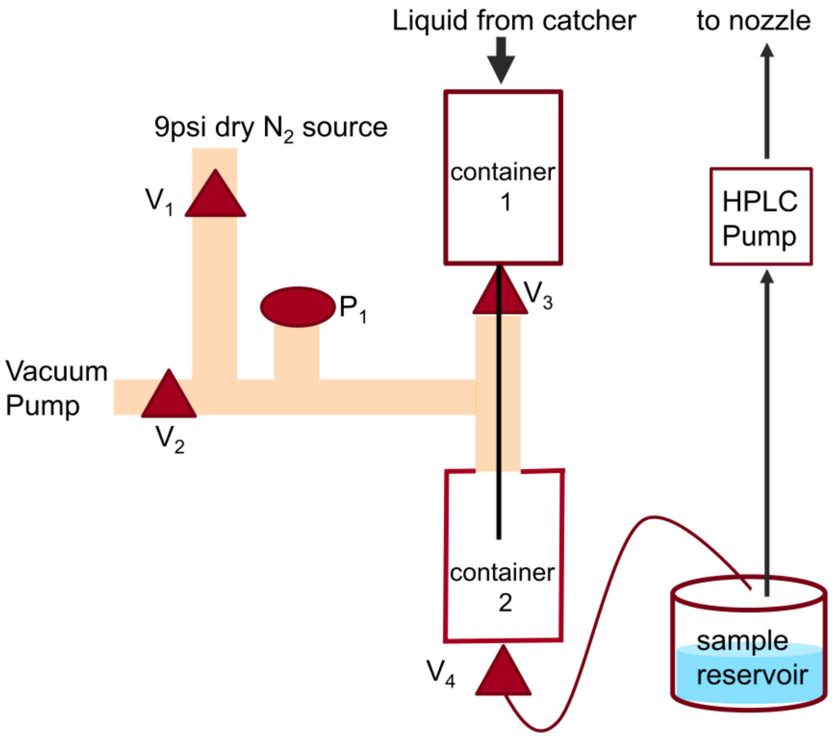

Supplementary Figure 3. Schematic diagram of the vacuum recirculation system. To start, both containers are at rough vacuum and all valves (V1-V4) are closed. P1 is a pressure gauge which monitors the pressure of container 2. Container 1 is chilled and held at vacuum and connected to a heated liquid catcher in the main vacuum chamber. Gravity and the vapor pressure gradient pushes liquid from the heated catcher to container 1. V3 is opened to allow for liquid to fall to the container 2 and then closed again. V1 and V4 are opened to backfill container 2 with nitrogen and push the liquid back to the reservoir. V1 and V4 are closed and V2 is opened to bring container 2 back to rough vacuum.
